# Characteristics, Symptom Severity, and Experiences of Patients Reporting Chronic Kidney Disease in the PatientsLikeMe Online Health Community: Retrospective and Qualitative Study

**DOI:** 10.2196/18548

**Published:** 2020-07-15

**Authors:** Glen James, Elisabeth Nyman, Marcy Fitz-Randolph, Anna Niklasson, Katarina Hedman, Jonatan Hedberg, Eric T Wittbrodt, Jennie Medin, Carol Moreno Quinn, Alaster M Allum, Cathy Emmas

**Affiliations:** 1 AstraZeneca Cambridge United Kingdom; 2 AstraZeneca Gothenburg Sweden; 3 PatientsLikeMe, Inc Cambridge, MA United States; 4 AstraZeneca Gaithersberg, MD United States; 5 AstraZeneca Luton United Kingdom

**Keywords:** community networks, chronic kidney disease, real-world experience, patient experience, retrospective, observational, diabetes, interview, online, social media

## Abstract

**Background:**

Chronic kidney disease (CKD) is a major global health burden, and is associated with increased adverse outcomes, poor quality of life, and substantial health care costs. While there is an increasing need to build patient-centered pathways for improving CKD management in clinical care, data in this field are scarce.

**Objective:**

The aim of this study was to understand patient-reported experiences, symptoms, outcomes, and treatment journeys among patients with CKD through a retrospective and qualitative approach based on data available through PatientsLikeMe (PLM), an online community where patients can connect and share experiences.

**Methods:**

Adult members (aged ≥18 years) with self-reported CKD within 30 days of enrollment, who were not on dialysis, and registered between 2011 and 2018 in the PLM community were eligible for the retrospective study. Patient demographics and disease characteristics/symptoms were collected from this retrospective data set. Qualitative data were collected prospectively through semistructured phone interviews in a subset of patients, and questions were oriented to better understand patients’ experiences with CKD and its management.

**Results:**

The retrospective data set included 1848 eligible patients with CKD, and median age was 56 years. The majority of patients were female (1217/1841, 66.11%) and most were US residents (1450/1661, 87.30%). Of the patients who reported comorbidities (n=1374), the most common were type 2 diabetes (783/1374, 56.99%), hypertension (664/1374, 48.33%), hypercholesterolemia (439/1374, 31.95%), and diabetic neuropathy (376/1374, 27.37%). The most commonly reported severe or moderate symptoms in patients reporting these symptoms were fatigue (347/484, 71.7%) and pain (278/476, 58.4%). In the qualitative study, 18 eligible patients (13 females) with a median age of 60 years and who were mainly US residents were interviewed. Three key concepts were identified by patients to be important to optimal care and management: listening to patient needs, coordinating health care across providers, and managing clinical care.

**Conclusions:**

This study provides a unique source of real-world information on the patient experience of CKD and its management by utilizing the PLM network. The results reveal the challenges these patients face living with an array of symptoms, and report key concepts identified by patients that can be used to further improve clinical care and management and inform future CKD studies.

## Introduction

Chronic kidney disease (CKD) is a major global health concern with an estimated prevalence of 8%-16% worldwide [1,2]. It is associated with an increased risk of adverse outcomes, poor health-related quality of life (HRQoL), and substantial health care costs [1]. The patient voice has become an integral part of the management of chronic diseases and increasingly relevant to the development of new treatments. Therefore, an expanding need exists to build patient-centered pathways for improved CKD management in clinical care [3]. Patient-focused research can help identify priorities and outcomes that are important to patients and can enable the development of care components that can improve health care practice [4-6]. This information has also driven the development of tools that can promote self-management, which has been shown to slow disease progression, specifically in patients with CKD [5,7]. While qualitative studies have identified specific topics of importance to patients with respect to disease management [4,5], there is still an outstanding need for information directly from patients about their experience of symptoms and outcomes in CKD [8-10].

More recently, the collection of data on the patient perspective of new treatments during drug development has been encouraged by the US Food and Drug Administration, which has developed guidance related to the use of patient‑reported outcome measures to support drug approvals and label claims [11]. Researchers in academia and industry are also increasingly seeking patients’ voices and candid perspectives on a variety of clinical research-related areas through patient advisory boards. These activities have generated valuable insights, leading to improvements in study design (eg, adjustments in visit schedules), patient communication materials (eg, study pamphlets), and the development of new patient-centered endpoints [12].

Patients with serious and chronic diseases increasingly seek information and peer-to-peer support through online communities [13-16]. Online health communities and research networks, such as PatientsLikeMe (PLM), allow patients to connect with other patients, share experiences, and learn more about how to live better with their condition [17,18]. Through their engagement with such communities, patients experience improved emotional and social well-being, as well as increased confidence in their interactions with health care providers, and greater optimism and control in relation to their condition [14,15]. Ultimately, these networks provide an opportunity for clinicians, service providers, and researchers to listen to and better understand patients, and gather information outside of the clinical setting. An additional method for eliciting in-depth information is through patient interviews. Qualitative studies involving semistructured interviews have been scarcely used to understand patients’ experiences of living with CKD and gain their perspective on various aspects of their care, so that any unmet needs can be identified and health care provision improved [4,10]. To date, characteristics, treatments, and self-reported outcomes in patients with CKD who are members of the PLM network have not been evaluated.

To improve patient care and to construct patient-centered pathways, it is necessary first to understand how patients experience their disease. The objective of this study was to understand patient-reported experiences, symptoms, outcomes, and treatment management among patients with CKD through a retrospective approach based on data available through PLM, and then using a qualitative approach in a subset of patients from the retrospective study cohort.

## Methods

### Retrospective Study Population

Adult PLM members (aged ≥18 years) were included in this study if they had registered with PLM between 2011 and 2018, if they had self-reported CKD within 30 days of registration, and if they were not receiving dialysis (N=1848).

### Qualitative Study Population

This population was a subgroup of the retrospective study cohort (acknowledging patients’ clinical profile, such as dialysis status, may have changed between enrollment on the retrospective study and the interview). All participants were adult PLM members (aged ≥18 years) who had self-reported CKD and were resident in the United States or other English-speaking countries (N=18).

### PatientsLikeMe

PLM, described in Multimedia Appendix 1, is an online network for patients with a range of diseases that allows them to connect with other patients and share personal stories and health data. Patients are directed to the website through multiple channels, including paid advertisements, public relations, press mentions, academic collaborations, word of mouth from other patients, provider referral, and web search [18]. Members provide information on their treatment and symptoms, including whether they are experiencing any core symptoms (pain, fatigue, anxious mood, depressed mood, and insomnia) that PLM asks members about regardless of their condition [19]. Participation is voluntary; patients did not receive an honorarium for providing data on the site, and their use of the site was not dependent on the provision of data. However, patients did receive an honorarium for interviews. Confidentiality of patient data was maintained and deidentified data were analyzed.

### Study Using Retrospective Data

A cross-sectional observational descriptive study using deidentified retrospective data from the PLM network database was used to characterize patients with self-reported CKD. These data were used under license for this study; restrictions applied to the availability of the data. Demographics and clinical characteristics were described for all patients who provided information. We evaluated data on CKD diagnosis, symptom severity (both general from the PLM symptom panel and other open-field symptoms), treatments, and comorbidities reported within 30 days of PLM registration. If multiple reports of the same symptom were entered within 30 days of registration, only the first report was included in these analyses. The 30-day period was chosen to reflect the status of patients when joining the PLM network. The data were standardized in PLM; comorbidities and symptoms were standardized to corresponding International Statistical Classification of Diseases and Related Health Problems, Tenth Revision codes, and treatments were standardized to prescription codes. Data were analyzed descriptively, presenting median with interquartile range (IQR), frequency, and proportion as appropriate. The study protocol was performed in accordance with International Conference on Harmonisation Good Clinical Practice and the Declaration of Helsinki, and the applicable legislation on noninterventional studies or observational studies or both. Patients who register on PLM are made aware that patient data will be used for research purposes, and results will be shared with the PLM community.

### Study Using Qualitative Data

The aim of this study was to understand the patient’s experience of CKD and its management. This involved questioning patients on their experience of CKD diagnosis, treatments, and comorbidities; the impact of symptoms on HRQoL and functioning; disease management, diet, and physical exercise in relation to CKD; and challenges around their interactions with their medical team, to better understand what is missing regarding specific CKD patient-reported management.

The qualitative study was performed in a subset of patients from the retrospective study using semistructured phone interviews (60-90 minutes). PLM members who met the inclusion criteria (ie, individuals who reported CKD as a condition, were over 18 years of age, and resided in the United States or in an English-speaking country) were identified and contacted by private message or email to participate. Those who indicated they wished to participate were contacted by the research team and any questions they had about the study were discussed. Consent forms were shared by email on scheduling, and verbal consent was obtained prior to starting the interview; all received US $100 for participating. Interviews were open ended and followed a semistructured interview guide. Open-ended questions were followed by prompts to encourage more detailed conversations about issues relevant to CKD if a topic had not already been fully explored. All interviews were recorded and transcribed postinterview to ensure direct quotes were accurate; recordings were deleted after they were transcribed verbatim.

Data were descriptively evaluated, presenting median with IQR, frequency, and proportion as appropriate. The interview transcripts were reviewed consecutively in groups of 3 using a content analysis approach [20] to identify emerging themes within the interview guide categories using the ATLAS.ti software (Scientific Software Development GmbH). As additional interviews were coded and new themes arose, the coding schema was updated, refined, and further grouped to create a final list of themes. This process was continued until no further concepts were identified and content saturation had been achieved (ie, no new concepts were being mentioned). Study end was achieved when either 20 participants had been interviewed or concept saturation was reached. Qualitative analyses of individual verbatim interview transcripts included whether symptoms reflected the patient experience without any potential bias that might be introduced by the interview questions [21]. The study protocol, patient communications, and interview guide were approved by the New England Independent Review Board. Any privacy concerns were addressed by not including identifying information in the final report and by allowing only authorized research personnel to access the PLM database.

### Data Availability

The data that support the findings of this study were collected from PatientsLikeMe. Restrictions apply to the availability of these data, which were used under license for this study.

## Results

### Retrospective Data

#### Demographics and Clinical History

Between 2011 and 2018, 1848 (from 2391) PLM members who self-reported CKD were not receiving dialysis and were included in this analysis (Multimedia Appendix 2). The majority of patients were female (1217/1841, 66.11%) and were US residents (1450/1661, 87.30%), with a median age of 56 years (IQR 45-64, n=1848) at the date they joined PLM (Table 1). Most of those who recorded race were white (1199/1496, 80.15%) and 79.1% (564/713) of patients who shared their education history had postsecondary education. The median age at onset of symptoms that patients associated with CKD was 43 years (IQR 28-54 years; n=378); median age of CKD diagnosis, reported by 578 members, was 47 years (IQR 33-56 years).

**Table 1:** Patient demographics and clinical history (retrospective study; total patients studied: 1848).

Characteristic			Total
**Gender (N=1841), n (%)**			
	Female		1217 (66.11)
Age (years) at date joined PLM^a^ (N=1848), median (IQR^b^); range			56 (45-64); 18-89
**Country (N=1661), n (%)**			
	United States		1450 (87.30)
	UK		66 (3.97)
	Other		145 (8.73)
**Race (N=1496), n (%)**			
	White		1199 (80.15)
	Black or African American		137 (9.16)
	Asian		67 (4.48)
	Mixed race		66 (4.41)
	Other^c^		27 (1.80)
**Education (N=713), n (%)**			
	Postgraduate degree (master’s, doctorate, etc.)		94 (13.18)
	College bachelor’s/undergraduate degree		136 (19.07)
	Some college, but less than a bachelor’s/undergraduate degree		334 (46.84)
	High-school graduate or GED^d^ (left school around age 18)		124 (17.39)
	Some high school, but did not graduate (left school around age 16)		23 (3.23)
	Eighth grade or less (left school around age 14)		2 (0.28)
Age (years) at first CKD^e^ symptom (N=378), median (IQR)			43 (28-54)
Age (years) at first CKD diagnosis (N=578), median (IQR)			47 (33-56)
Time (months) between first symptom and diagnosis (N=371), median (IQR)			0.0 (0.0-12.0)
Time (months) between first symptom and joining PLM (N=378), median (IQR)			63.9 (28.9-9.3)
Time (months) between diagnosis and joining PLM (N=578), median (IQR)			40.9 (14.5-102.3)
**Medication (N=1369), n (%)^f^**			
	**Diabetes medication**		702 (51.28)
		Metformin^g^	481 (68.52)
	**Hypertension medication**		311 (22.72)
		Angiotensin-converting enzyme inhibitor^g^	212 (68.17)
	**Antidepressant medication**		301 (21.99)
		Serotonin–norepinephrine reuptake inhibitors^g^	150 (49.83)
	**Lipid-modifying medication**		267 (19.50)
		Statin^g^	247 (92.51)
	**Immunosuppressants**		147 (10.74)
		Calcineurin inhibitors^g^	30 (20.41)
	**Anti-inflammatory and antirheumatic medication**		52 (3.80)
		Nonsteroidal anti-inflammatory drugs^g^	48 (92.31)

^a^PLM: PatientsLikeMe.

^b^IQR: interquartile range.

^c^Includes American Indian or Alaska Native, or Native Hawaiian or other Pacific Islander.

^d^GED: General Educational Diploma.

^e^CKD: chronic kidney disease.

^f^Percent for medication class is percent of those reporting treatment (N=1369), whereas percent for top reported medication within treatment class is percent of treatment class.

^g^Top reported medication within the specified drug class.

#### Comorbidities and Treatments

**Figure 1:**
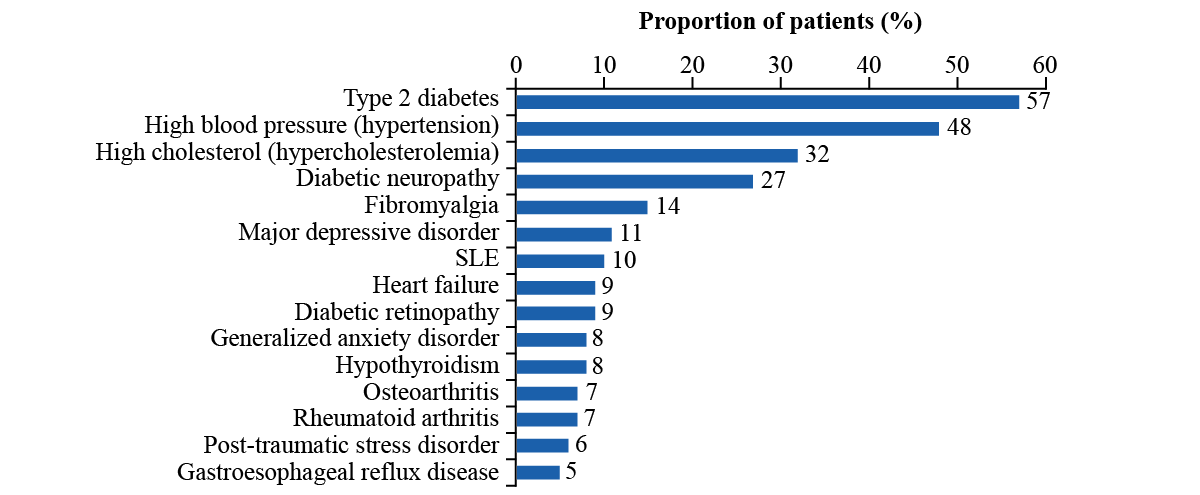
Patient-reported comorbidities (retrospective data). Comorbidities reported in addition to CKD (n=1374). CKD: chronic kidney disease; SLE: systemic lupus erythematosus.

Conditions in addition to CKD were reported in 1374/1848 patients (74.35%), and the median number of different conditions per patient was 3 (IQR 0-4). There were 474 patients reporting no comorbidities, 685 patients reporting 1-3 comorbidities, and 689 patients reporting more than 3 comorbidities. The most common conditions among those reporting comorbidities were type 2 diabetes (783/1374, 56.99%), hypertension (664/1374, 48.33%), hypercholesterolemia (439/1374, 31.95%), and diabetic neuropathy (376/1374, 27.37%; Figure 1). Treatments were reported by 1369 patients; the most common were for diabetes (702/1369, 51.28%) and hypertension (311/1369, 22.72%; Table 1). These comorbidities are highly prevalent in patients with CKD.

#### Symptoms

**Figure 2:**
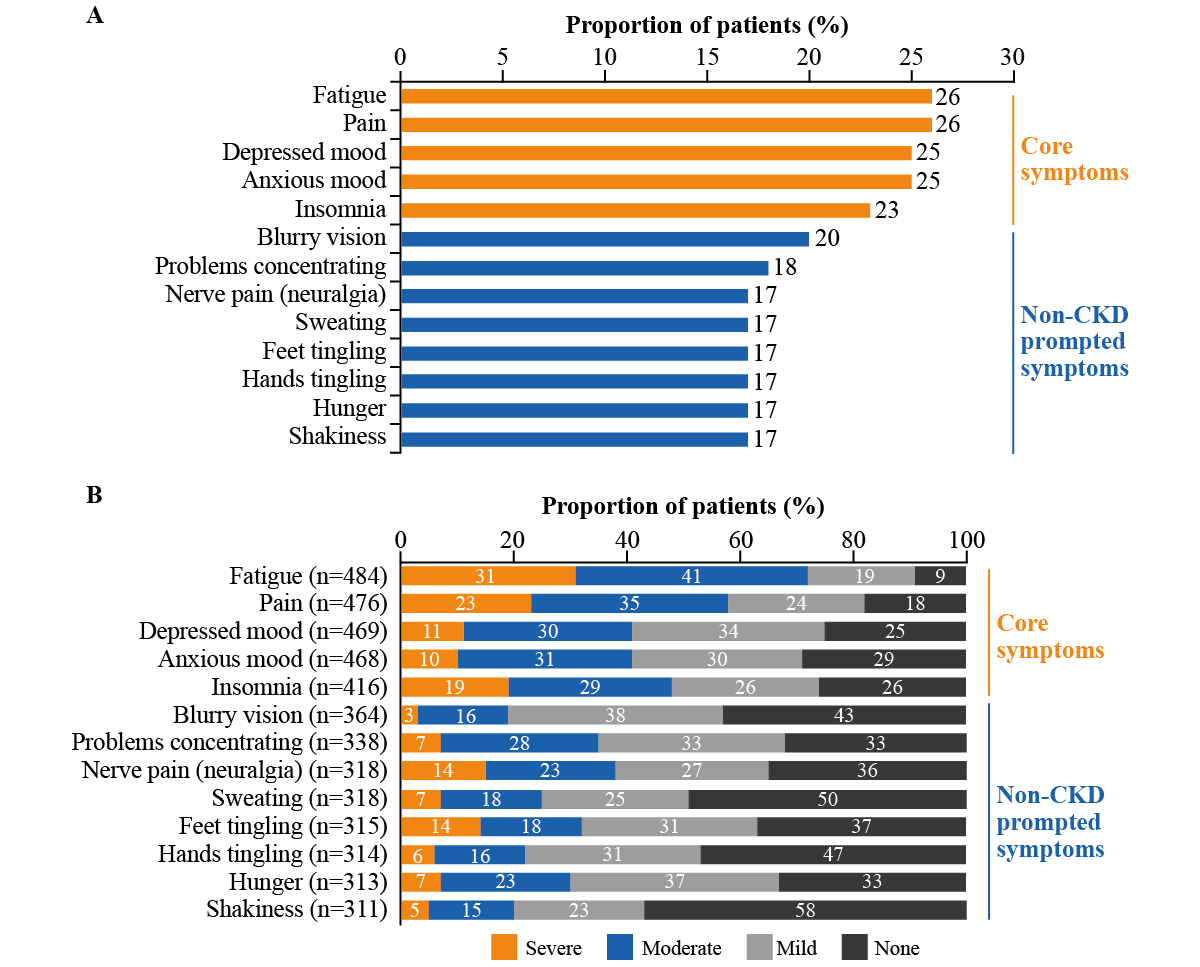
Patient-reported symptoms (retrospective data). (A) Core and additional patient-reported symptoms (total cohort, N=1848). (B) Severity of patient-reported symptoms. Core symptoms are symptoms that all patients registered with PatientsLikeMe are asked to track; additional symptoms are those patients themselves may choose to track, or symptoms patients may be asked about in relation to a condition other than CKD. *None* refers to those patients reporting *none* for a specific symptom; those who did not provide any response were not included. CKD: chronic kidney disease.

Less than one-half of all patients (754/1848, 40.80%) entered any symptom within 30 days of registration. Core symptoms from the PLM panel were reported by 487 patients (26.35%), and 633 patients (34.25%) reported additional symptoms (Figure 2A). Core symptoms of fatigue, pain, and insomnia were rated as moderate or severe by 71.7% (347/484), 58.4% (278/476), and 47.6% (198/416) of patients, respectively (Figure 2B). Of the additional symptoms, nerve pain, problems concentrating, and feet tingling were classified as moderate or severe by more than 30% of patients—37.1% (118/318), 34.6% (117/338), and 32.4% (102/315), respectively.

### Qualitative Semistructured Interviews

Concept saturation (Multimedia Appendix 3) was reached with 18 patients, after which no additional patients were interviewed. In-depth patient perspectives on their disease and care were gathered during these semistructured interviews and 4 major concepts were explored: symptoms, financial burden, valued outcome, and care concepts. We explore the interview responses within these themes below.

#### Patient History With CKD, Including Diagnosis

This group of 18 patients had a median age of 60 (IQR 52-76) years, were mostly US residents (n=16), and female (n=13). At the time of the interview, 2 patients who had participated in the retrospective study had stage 4 CKD and were receiving or about to initiate dialysis. Most patients described their current health status as *OK* (n=7) or *Good* (n=8). The median patient age at diagnosis of CKD was 55 (IQR 47-62) years and 15 patients were able to report disease stage at diagnosis (Table 2). Most patients explained that they were diagnosed by a nephrologist following referral by their primary care physician (Textbox 1), and their self-reported diagnosis was based on an abnormal metabolic panel or urinary protein on screening tests.

All patients had comorbidities, with the most common being hypertension (12/18, 67%; Table 2). These comorbidities also had symptoms that could be associated with CKD, especially fatigue, numbness/tingling, or nausea.

**Table 2:** Patient demographics (qualitative study).

Characteristic		Total (N=18)
Age (years), median (interquartile range)		60 (52-76)
Female, n (%)		13 (72)
**Location, n (%)**		
	United States	16 (89)
	Ex-United States	2 (11)
**Work status,** **n (%)**		
	Full-time	1 (6)
	Part-time	3 (17)
	Retired	4 (22)
	Disabled^a^	9 (50)
	Homemaker	1 (6)
**Chronic kidney disease stage at diagnosis,** **n (%)**		
	Unknown	3 (17)
	2	2 (11)
	3a/3b	11 (61)
	4	2 (11)
**Most frequently reported comorbidities,** **n (%)**		
	Hypertension^b^	12 (67)
	Osteoarthritis/degenerative joint disease related	9 (50)
	Gastroesophageal reflux disease and related conditions	7 (39)
	Anemia^b^	7 (39)
	Fibromyalgia/myofascial pain	6 (33)
	Peripheral neuropathy	6 (33)
	Diabetes^b^	4 (22)
	Hyperkalemia^b^	3 (17)

^a^Disability not due to chronic kidney disease.

^b^These comorbidities were probed for if not spontaneously described by patients.

**Direct patient quotes from the qualitative study. Patients provided consent to include their words in a deidentified format. CKD: chronic kidney disease.**

**Patient history with CKD, including how they were diagnosed**

At one time I was on so many medications that it was ridiculous, and I’ve got off of everything except some Parkinson’s medicine and blood pressure medicine and blood thinner that I have to take.

I discovered I had chronic kidney disease when I was participating in the dry eye study. During the study I was asked to provide a urine sample, which showed protein in my urine.

And she says, and another thing, she says, we’re going to have to send you a specialist on is that you’re showing signs of kidney disease, and she says we’re looking at stage 3.

I had just had some routine lab work, and it showed that my eGFR was low consistently. He repeated it a few times. So they sent me to a nephrologist.

I never am really sure what’s contributing to it, if the kidney disease is a part of that, especially my hemoglobin. I don’t get anemic or anything how that fatigue really sets in. I usually attributed it to the fibromyalgia.

**Patient symptom experience related to CKD**

I could spit sand.

I’m doing a workup for a kidney transplant also, so that’s why I had the day off today, and then just like this last month I’ve had like six different doctors’ appointments.

I did have very severe anemia. I had many, many blood transfusions.

**Financial impact of CKD**

Well, I don’t have the best insurance in the world, but so far I’ve been able to pay most of my bills.

When I was finally approved for disability, I was also approved for (inaudible) Medicare. So that helped a bunch. Because basically, I had to stop working in 2014 because of the health issues.

I’d say most of my problems are financial right now. So now it’s taking the time off plus the drugs plus – it’s just the whole situation and everything.

It can run up to $700 a month with MRIs, CT scans, doctor bills. It’s been bad lately.

**CKD patient experience with diet and physical exercise**

Yeah, there are times when I can’t really go out or do anything even at home, because my muscles are so cramped up.

Losing weight, trying to exercise regularly.

Not really happy [about my diet] because I grew up on a farm and meat was the main – we had meat three times a day, and I’m a real meat eater. So that has been a huge change for me.

And they keep always saying you got – you have to exercise. The extra weight is hard on the kidneys. And I don’t know what my problem is. It’s a lack of motivation, I think.

He always talks to me about doing what I can. Don’t overdo it, obviously ... Yeah, he tries to keep me motivated and at least doing some sort of exercise daily, even if just like for 10 or 15 minutes, whatever, to keep my body moving.

**Patient perception of CKD care and management**

... They didn’t honestly seem very concerned about my kidney health as much.

But I was always having to remind everybody, hey, I’ve got a kidney problem. We have to keep that in mind that it’s not always the pulmonary hypertension. It’s not always the small fiber neuropathy. So, I have to make sure that they understand that.

I want to have the best life that I can, and if I can’t take anything for pain because of my kidneys, my life isn’t going to be the best life I have. So, I have to know what things will work for me but aren’t going to continue damaging my kidneys.

He’s just very understanding, very supportive, and he does a good job of explaining what’s going on. He gives good praise for achieving results, which helps.

I think at this point it’s me. That’ll change, probably. But I’m pretty independent, and I want to be in control. I think most people do.

... so, I have learned that I don’t ask him my questions. I ask my nephrologist my questions. And he will answer anything. And he wants to answer. But the primary doctor just doesn’t seem to care.

For the record, I would never have another transplant – and God hope my kidneys don’t go south, because I would never do it again. Never in a million years.

Shifting to a new one is a little bit scary.

You really need to have a doctor that’s going to really listen to you because he’s going to miss something if he’s not listening to you.

Well, I just hope that I can live the rest of my life and not have serious problems with my kidney.

I just make sure that I speak up for myself and explain where I’m at or ask questions. I think that that’s a key component that patients don’t always do. If nothing else, nursing has taught me to be an advocate for myself.

Someone’s taking care of it, and I know that I will know if my kidney function starts to fall or something, they’ll know, and they’ll start to get it taken care of right away.

It’s just that nagging thought back there about well, will I be able to continue to live the quality of life that I do, and my kidney function remain high enough that I don’t need more aggressive treatment?

But the doctors that I have here I feel are great doctors, and they really work with me ... So, they all have to be working in concert, which I’ve worked really hard to get them to do.

#### Patient Symptom Experience and HRQoL

**Figure 3:**
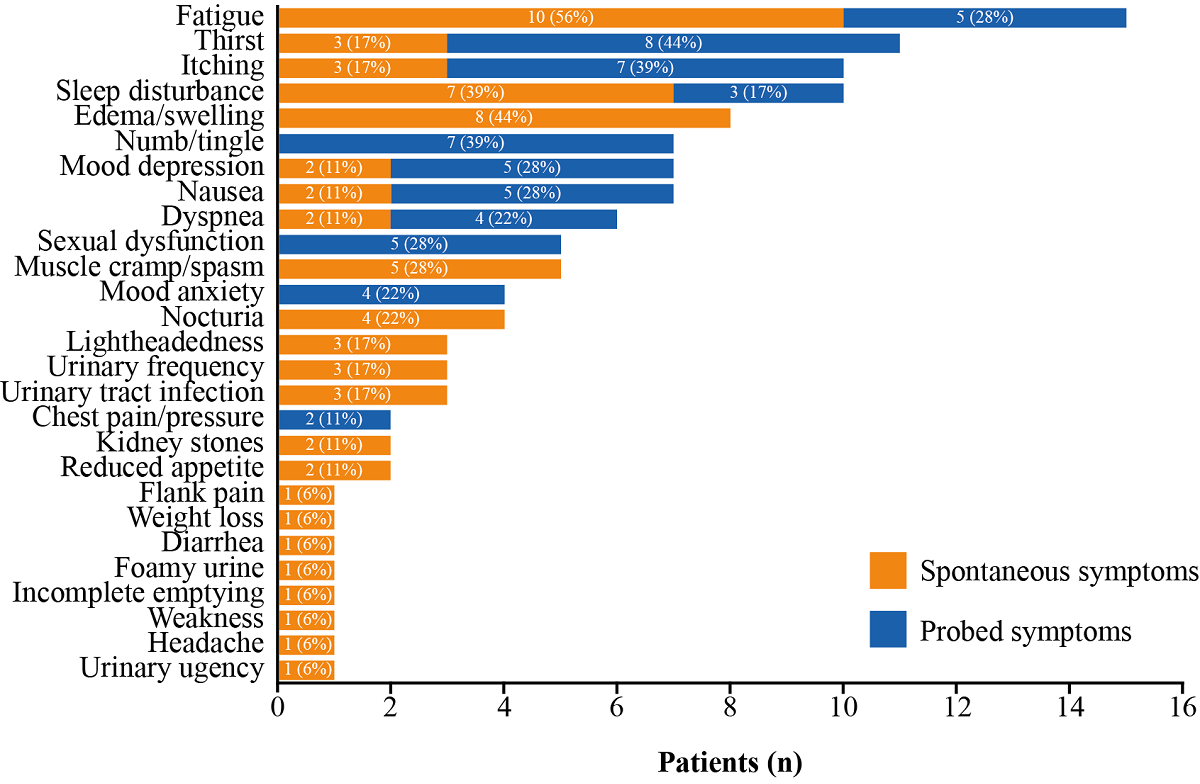
Patient-reported symptoms (qualitative study, N=18). Spontaneous symptoms are symptoms that patients themselves choose to share during the interview that they considered to be related to CKD, probed symptoms are those that interviewers probed for if patients did not mention them (fatigue, sleep disturbance, itching, numbness/tingling, chest pains, dyspnea, nausea, thirst, mood, and sexual function). If a patient had not mentioned any issues related to sleep spontaneously, they were asked about “any impacts on their sleep”; similarly, if they had not mentioned any issues related to anxiety or depression, they were asked “how does CKD affect your mood?”. Uniformly, patients reported negative impacts on sleep, mood, and sexual functioning either spontaneously or prompted.

In total, 28 different symptoms were reported by patients as potentially related to CKD (eg, edema, urinary tract infection, and urinary frequency). Textbox 1 summarizes key quotes from patients. The most commonly reported symptoms (spontaneous and probed) included fatigue (n=15), thirst (n=11), itching (n=10), sleep disturbance (n=10), and edema/swelling (n=8) (Figure 3). Edema/swelling and muscle cramps/spasms were 2 commonly reported symptoms not probed for during interviews. These symptoms also influenced both social interactions and the activities of daily living. For example, 1 patient noted that their body would suddenly *go rubbery* on them during yard work (Textbox 1).

When asked which symptom had the biggest impact on their HRQoL, most patients (n=12) mentioned a symptom not related to CKD or said no symptoms had a significant impact. Of the CKD-related symptoms mentioned as having the biggest impact, fatigue was most commonly reported (n=4), followed by muscle cramping (n=2). Six patients reported experiencing fatigue *all of the time*, whereas 2 patients commented that CKD affected their sleep *all of the time* (Multimedia Appendix 4), and the impact of lack of sleep on fatigue was noted.

Only the 2 patients with end-stage renal disease (ie, stage 4 or 5, on or preparing for dialysis) stated that CKD was the single biggest health burden in their lives. One patient commented that bipolar disorder and CKD equally caused the most burden; another said diabetes and CKD. Most of the remaining patients ranked CKD third or lower in their list of comorbidities.

#### Financial Impact on Patients

Nine patients reported a financial impact, with 4 patients spending *some* and 3 patients spending *a lot* of their monthly budget toward medical bills; 2 patients noted that only *a little* was related to medical bills. The level of financial impact varied across patients and was influenced by insurance status, severity of CKD, and comorbidities. The financial impact of CKD was highlighted by 12 patients in total (Multimedia Appendix 4). Concepts appearing when describing financial impact of CKD included concern about loss of insurance, high deductibles, insurance dictating care, losing time at work leading to lost wages, expensive drugs, cost of specialists, and time and knowledge required to scrutinize billing (Textbox 1).

#### Patient Experience With Diet and Physical Exercise

Physical activity was considered important; however, nothing more than regular exercise or walking for CKD was prescribed (Textbox 1). Some patients with comorbidities did report they received physical therapy, and it was clear that physicians were actively encouraging patients to engage in physical activity.

A total of 7 patients shared that they had received recommendations for physical activity from clinicians, but all instances were very general and not specific for CKD. Patients received more information about diet monitoring than about physical activity, because they discussed diet details when reviewing their course of treatment for CKD. Almost all patients mentioned monitoring aspects of their diet, such as salt, potassium, protein, and water intake.

Many patients noted that they missed foods they like, such as pasta, but accepted diet changes. However, it was clear that for some patients, balancing diet changes with multiple conditions was challenging. Vitamin and mineral supplement use were reported, specifically when iron-deficiency anemia or hypokalemia were mentioned.

#### Disease Management

When asked what they valued in managing their CKD, most patients focused on the reduction of future harm (ie, minimizing kidney damage, preventing disease progression, and not losing kidney function; Textbox 1). For example, 1 patient recognized the importance of preserving kidney function: “I want to have the best life that I can, and if I can’t take anything for pain because of my kidneys, my life isn’t going to be the best life I have. So, I have to know what things will work for me but aren’t going to continue damaging my kidneys.” Another patient viewed their HRQoL as the most important aspect of care: “Quality of life is more important to me than length of life.” Other concepts valued by patients included awareness of CKD by all their doctors, a firm diagnosis, regular monitoring, having future treatment choices (dialysis, transplant, or none) honored, preferring transplant to dialysis, and having a normal life (Textbox 1). Importantly, one patient summarized that “I want to be able to make my own choices, and if I decide that I go on dialysis, I want support for that. If I don’t want to, I want support for that, too”.

**Figure 4:**
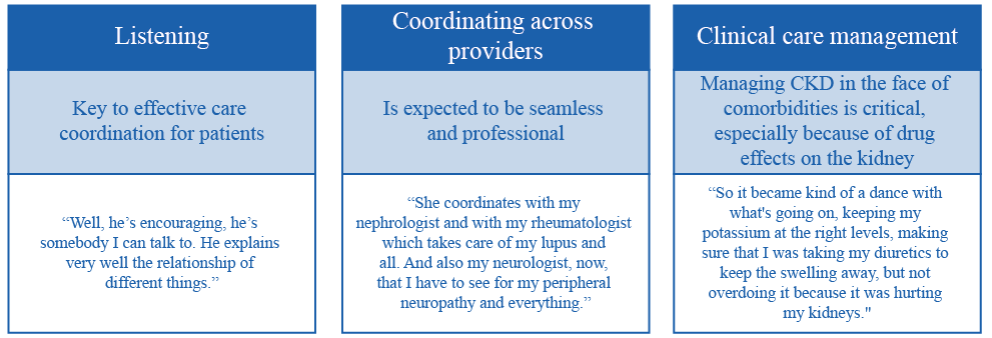
Concepts related to optimal CKD care and management (qualitative study). CKD: chronic kidney disease.

Patients with CKD require coordination of care with their primary care physician, renal specialists, and specialists managing their comorbidities. Most patients listed their primary care physician, psychiatrist, and nephrologist as the most helpful in managing their CKD; 4 patients said they themselves were most helpful (Textbox 1). Patients frequently commented on the aspects of their care that led to well‑coordinated, effective CKD management. Concepts generated by patients fell into 3 overarching domains: listening, coordinating across providers, and clinical care management (Figure 4). Listening to patients’ needs was considered central to effectively coordinating care. There was an expectation among patients that coordinated care across providers should be seamless to ensure appropriate management of their CKD in the face of comorbidities. When any of these aspects of care were absent, patients felt these were unmet needs (Textbox 1).

## Discussion

### Principal Results

The patient voice is often underrepresented in the clinical setting, yet it is critical to optimizing and improving clinical practice and research, and to better understand chronic conditions such as CKD. Our main objective in this study was to better understand the patient experience with CKD and its management through direct patient insight, unfiltered by interactions with health care professionals, from a large patient cohort within the PLM network. This study highlighted the array of symptoms that patients with CKD face, as well as key concepts that are important for optimal care and management.

To our knowledge, this is the first published analysis of real-world data gathered on CKD from an online community. Data from PLM have previously been used to explore patient perspectives on neuromyelitis optica and neuromyelitis optica spectrum disorders [19], characterize the profiles of patients with atopic dermatitis [22] and systemic lupus erythematosus [23], and understand patient preferences for type 2 diabetes self-management [24]. This is the first study to utilize the PLM network to gain insights into self-reported experiences in patients with CKD. These approaches for data collection can also translate to the clinical trial design process. Engaging patients early in the process has the potential to ultimately yield more successful studies [25]. DISCOVER CKD is an example of a study that has been influenced by this patient-centric approach (ClinicalTrials.gov identifier: NCT04034992). This PLM study was the pilot study for developing the interviews used in the DISCOVER CKD study and guided decisions about which symptoms the DISCOVER CKD study should capture [26].

### Retrospective Study

The retrospective study profiled a specific cohort of PLM members with CKD who were not receiving dialysis. Their characteristics are broadly consistent with US CKD populations from United States Renal Data System (USRDS), National Health and Nutrition Examination Survey (NHANES), and the Dialysis Outcomes and Practice Patterns Study (DOPPS), although the percentage of females is slightly higher [27-29]. Members of PLM with CKD included in this cohort were predominantly white, middle‑aged (median 56 years), and women, whereas the prevalence of CKD is only slightly higher for women compared with men (17% vs 13%, respectively) in the general population, with the majority aged >60 [27]. PLM members also have a higher educational level than the general population with CKD; 79.1% (564/713) had postsecondary education compared with 56%-59% in the general population [27,28]. The overrepresentation of educated men and women in the cohort and the relatively young age of PLM members compared with the general population according to the references reflect the patient population who regularly use health-based internet sites [30,31].

Comorbidities were reported by 74.35% (1374/1848) of the PLM cohort studied, the most common being type 2 diabetes (783/1374, 56.99%), hypertension (664/1374, 48.33%), hypercholesterolemia (439/1374, 31.95%), and diabetic neuropathy (376/1374, 27.37%). This is reflective of the general CKD population, as reported by the USRDS, where diabetes (36%), hypertension (31%), and self-reported cardiovascular disease (40%) are the most common comorbidities [27]. While rates of major depressive disorder (148/1374, 10.77%) were close to the frequency of depressive symptoms among predialysis patients (13%) attending a single renal center [32], they were lower than the reported prevalence of depressive symptoms among patients with CKD who are not on dialysis (21%-42%) [33]. The proportion of patients with fibromyalgia (199/1374, 14.48%) was higher than that previously reported in patients with CKD receiving hemodialysis (3.9%-12.2%) [34-37] or peritoneal dialysis (9.7%-18%) [38,39]. This may be due to the nature of the PLM database, where large communities for chronic conditions including fibromyalgia exist [17]. Therefore, these comorbidities tend to be overrepresented. Overall, the similarities between the characteristics of the PLM network and the overall US CKD population indicate that the PLM platform is a useful format for gathering insights into CKD care and management.

### Qualitative Study

Information gathered from semistructured interviews with a small subset of the retrospective study population provides a unique in-depth perspective on patient experiences and captures their opinions regarding the care they receive or gaps they perceive in their care, including insights that may not be collected in a routine clinical setting or captured by other patient‑reported outcome tools. Four key concepts were explored in the interviews: symptoms, financial burden, valued outcome, and care concepts.

Patients shared how their symptoms affected their HRQoL, with fatigue noted by most patients as having the biggest impact. Fatigue and insomnia were also among the top core symptoms reported in the retrospective population. Together, these findings highlight the significance of fatigue and sleep-related problems for patients, corroborating other CKD studies [40-43]. Patients with CKD are known to experience profound fatigue and it is one of the most common symptoms affecting their HRQoL [43,44]. One study reported how fatigue was associated with unemployment, comorbidities, and use of antidepressant medication; the authors suggested that the presence of fatigue may act as a clinical prognostic factor in CKD [45]. Patients experiencing sleep disorders and fatigue are also at increased risk of all-cause mortality [44]. Fatigue has also been related to poor physical functioning, although causality remains unclear [41].

The cause of fatigue experienced by this subset of PLM patients is likely to be multifactorial [41] and associated with prevalent complications such as anemia [41,45] and low albumin levels [42], psychosocial factors including depression and anxiety [41], and insomnia [46]. Improved understanding of the impact that fatigue and other symptoms such as depression and anxiety have on those living with CKD can help guide treatment and improve quality of care by prioritizing interventions that improve HRQoL, such as personalized care, precision medicine, or wearable technology and biometric devices.

Only the 2 patients with stage 4 CKD considered CKD to be their single biggest health burden. This finding is likely due to the predominance of earlier and less symptomatic stages of CKD (3a or 3b) in this study and to the nonspecific nature of CKD-related symptoms. Other patients identified other comorbidities (eg, diabetes) as more burdensome relative to CKD. These comorbidities are interrelated with CKD and one can affect another; therefore, awareness among patients regarding which symptoms are associated with specific diseases needs to be addressed in the clinical management of patients with CKD.

Financial concepts around loss of insurance and earnings, and the cost of treatment and specialist care arose from interviews. Importantly, patients’ experiences of the financial impact of CKD were varied and influenced by insurance status (particularly in regions without universal health care), severity of CKD, and other comorbidities. According to the USRDS, total medical costs were US $50.4 billion for CKD (excluding end-stage renal disease) among Medicare patients in 2013 [47]. In adult patients in the United States, direct annual health care costs for individuals with CKD were US $17,472 higher compared with those without CKD from 2002 to 2011 [48]. Such financial pressures experienced by patients may impact other symptoms noted, such as depression [49], and should therefore be considered in the holistic management of patients in clinical care.

The personal experiences of some interviewees reflected but were not fully aligned with the current Kidney Disease: Improving Global Outcomes (KDIGO) guidelines [50,51]. Current guidelines recommend that those living with CKD should be encouraged to undertake physical exercise, achieve a healthy weight (BMI 20-25 kg/m^2^), and stop smoking. KDIGO guidelines state that expert dietary advice and information should be provided to patients in the context of an education program and tailored to severity of CKD. Detailed recommendations on protein intake, glycemic control, and salt and potassium intake are also provided [50,52]. However, PLM patients in this study mentioned that any lifestyle advice they received was very general and they were more likely to receive information about diet than physical activity; almost all patients noted that they monitored aspects of their diet. Importantly, some patients found balancing diet changes with multiple conditions very challenging. These insights illustrate that KDIGO guidelines on exercise may not be implemented effectively in daily practice and patients with multiple conditions need additional support from health care providers to manage the changes in their diet.

Concepts identified by patients relating to optimal CKD care and management fell into 3 domains: listening, coordinating across providers, and clinical care management. Patients valued physicians who listened to their needs, coordinated their care effectively with other specialists, and managed their CKD in conjunction with comorbidities. When any of these aspects of care were absent, patients felt these were unmet needs. These differ slightly to The Renal Association and Kidney Care UK’s Kidney Patient Reported Experience Measure, a UK initiative to gain insight into patient experiences and help shape renal services, which identified 3 aspects of renal care that affect patient experience: shared decision making, transport, and discomfort associated with needle insertions [53]. Such differences may reflect differences in health care systems between countries, such as the National Health Service in the UK versus an insurance-based system in the United States. These insights provide an opportunity for health care service providers to adapt local clinical practice to improve care and better reflect the experiences and needs of patients. Patient experience has been positively associated with patient safety and health outcomes, and clinical effectiveness across a number of diseases and settings [54].

### Study Limitations

A limitation of this study was that members of PLM self-reported their diagnoses and treatment—there was no independent clinical corroboration; and treatment indication, although captured by PLM, was not analyzed as few patients provide this information. In addition, the extent and nature of missing data can reduce precision and lead to a systematic bias of results; however, missing variables and missingness in variables were assessed and reported. Because PLM members were not expected to know details of their disease severity (ie, CKD stage), it is also not possible to derive information on how self-management and care needs to change with disease progression.

There is also a sampling bias as patients had an active choice to participate/enroll into the PLM network, so the cohort was self-selecting. As a result, generalizability of these results may be limited because the profiles of patients enrolling into the PLM network may differ from the profile of those not enrolling into PLM or of the general population. The observation that patients who enroll in PLM with CKD tend to be younger, more likely to have some college education, and more likely to have white ethnicity compared with the general population has also been noted in other disease groups [19,55].

The evaluation of the retrospective data in this study focused on information reported within 30 days of enrollment/registration (or as close as possible) into the PLM network. However, we cannot be sure all information reported during or outside this period relates specifically to CKD, as most patients reported multiple comorbidities. There may be a future opportunity to conduct a longitudinal study to describe patient experience over time.

Nonetheless, in the retrospective study, the use of data directly from an activated network of patients that were not filtered through an interviewer or clinician eliminated any potential interviewer bias and the use of an anonymous online survey may have improved willingness to disclose sensitive information.

### Conclusions

This study provides a unique source of real-world information on the patient experience of CKD and its management by utilizing the PLM network. The data collected through PLM identified key symptoms affecting patients, including fatigue and depression/anxiety, and are broadly consistent with published data on CKD populations in the United States. In-depth semistructured interviews with members reveal the challenges patients face living with symptoms of CKD and the impacts they experience financially and on HRQoL. These interviews identified key concepts around optimal care and management of CKD such as listening to patients and coordinating health care across providers, indicating that this approach can provide insights outside of the clinical setting to inform clinical practice and drug development, and improve future studies.

## Appendix

Multimedia Appendix 1 Supplementary material - Methods.

Multimedia Appendix 2 Patient disposition (retrospective data).

Multimedia Appendix 3 Concept saturation (qualitative study).

Multimedia Appendix 4 Patient-reported symptoms (qualitative study, N=18).

## Abbreviations

CKD: chronic kidney disease
HRQoL: health-related quality of life
IQR: interquartile range
PLM: PatientsLikeMe
