# Supplementary material for: Characteristics, Symptom Severity, and Experiences of Patients Reporting Chronic Kidney Disease in the PatientsLikeMe Online Health Community: Retrospective and Qualitative Study
Source: J Med Internet Res. 2020 Jul 15;22(7):e18548. doi: 10.2196/18548 (PMC7391670; doi:10.2196/18548)
Supplement: Multimedia Appendix 1 [file jmir_v22i7e18548_app1.docx]

# Multimedia Appendix

**Characteristics, Symptom Severity, and Experiences of Patients Reporting Chronic Kidney Disease in the PatientsLikeMe Online Health Community: Retrospective and Qualitative Study**

Glen James^1^, PhD; Elisabeth Nyman^2^, MSc; Marcy Fitz-Randolph^3^, DO, MPH; Anna Niklasson^2^, PhD; Katarina Hedman^2^, PhD; Jonatan Hedberg^2^, MSc; Eric T. Wittbrodt^4^, PharmD, MPH; Jennie Medin^2^, MPH, PhD; Carol Moreno Quinn^1^, MD, PhD; Alaster M. Allum^1^, MBBS, BA, DipSI; Cathy Emmas^5^, PhD

^1^AstraZeneca, Cambridge, UK

^2^AstraZeneca, Gothenburg, Sweden

^3^PatientsLikeMe, Inc., Cambridge, MA, USA

^4^AstraZeneca, Gaithersburg, MD, USA

^5^AstraZeneca, Luton, UK

## Multimedia Appendix 1

## PatientsLikeMe

The PLM network encourages patients to track their symptoms, treatments, side effects, and outcomes to improve their own healthcare and to advance medical research into their conditions [50]. As of 2018, most of the 685 464 total members are based in the USA (~81%), UK (~6%), and Canada (~3%), and have reported over 2900 conditions. Out of the total number of members, 2391 patients with CKD were registered on PLM as of December 31, 2018, of which ~300 were active on the network, ie, had any activity on the site in the previous 90 days.
