# Supplementary material for: Characteristics, Symptom Severity, and Experiences of Patients Reporting Chronic Kidney Disease in the PatientsLikeMe Online Health Community: Retrospective and Qualitative Study
Source: J Med Internet Res. 2020 Jul 15;22(7):e18548. doi: 10.2196/18548 (PMC7391670; doi:10.2196/18548)
Supplement: Multimedia Appendix 3 [file jmir_v22i7e18548_app3.docx]

Multimedia Appendix 3. Concept saturation (qualitative study)


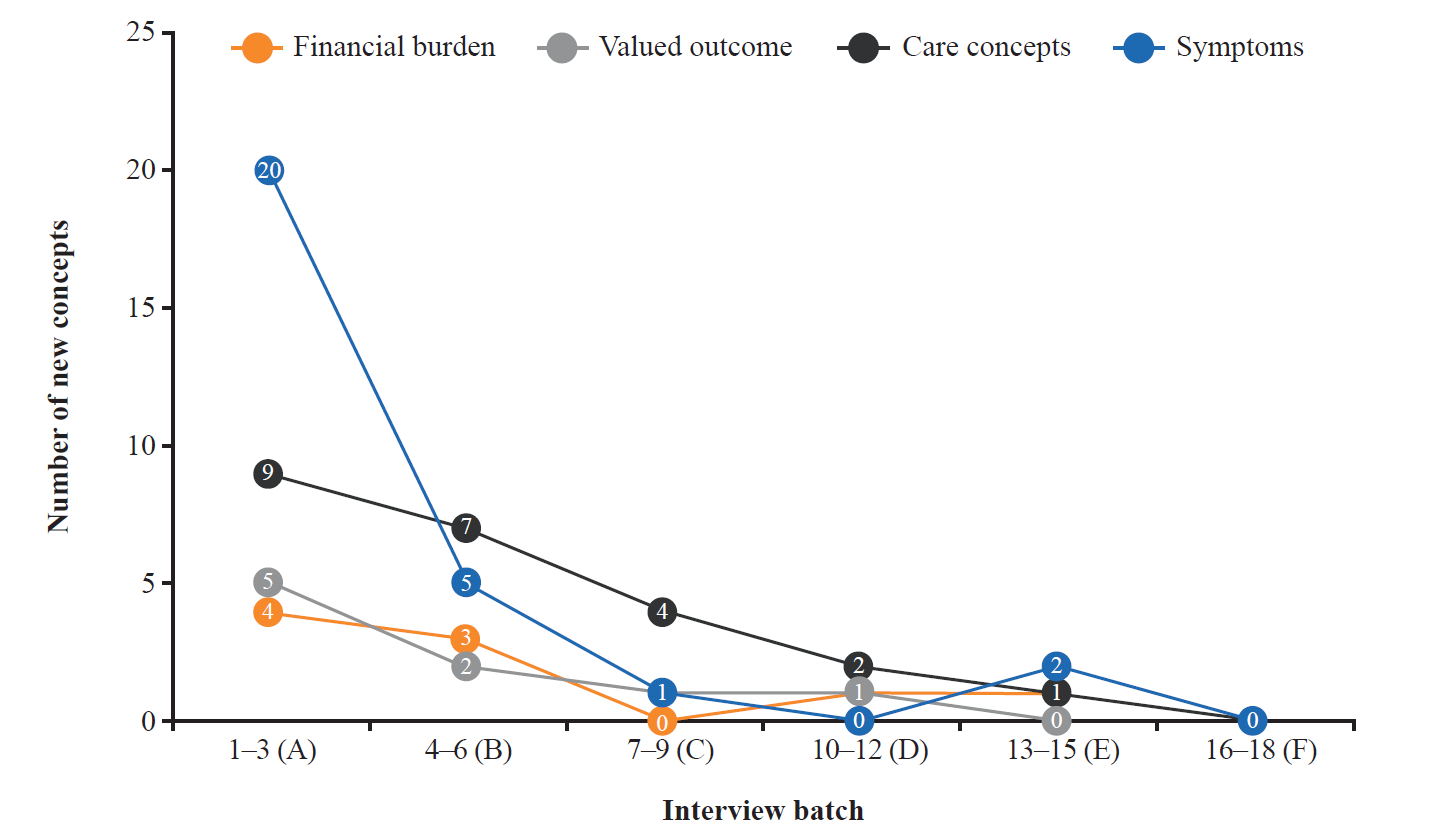


Interviews were divided into 6 cohorts (A–F) of 3 interviews each. For each area discussed, the number of new concepts identified in the cohort is shown on the graph. “Valued Outcome” saturated at cohort E, while the rest saturated at cohort F. By the final cohort, no new concepts were appearing in any area, indicating concept saturation.
