# Supplementary material for: Characteristics, Symptom Severity, and Experiences of Patients Reporting Chronic Kidney Disease in the PatientsLikeMe Online Health Community: Retrospective and Qualitative Study
Source: J Med Internet Res. 2020 Jul 15;22(7):e18548. doi: 10.2196/18548 (PMC7391670; doi:10.2196/18548)
Supplement: Multimedia Appendix 4 [file jmir_v22i7e18548_app4.docx]

Multimedia Appendix 4. Patient-reported symptoms (qualitative study, N=18)

**
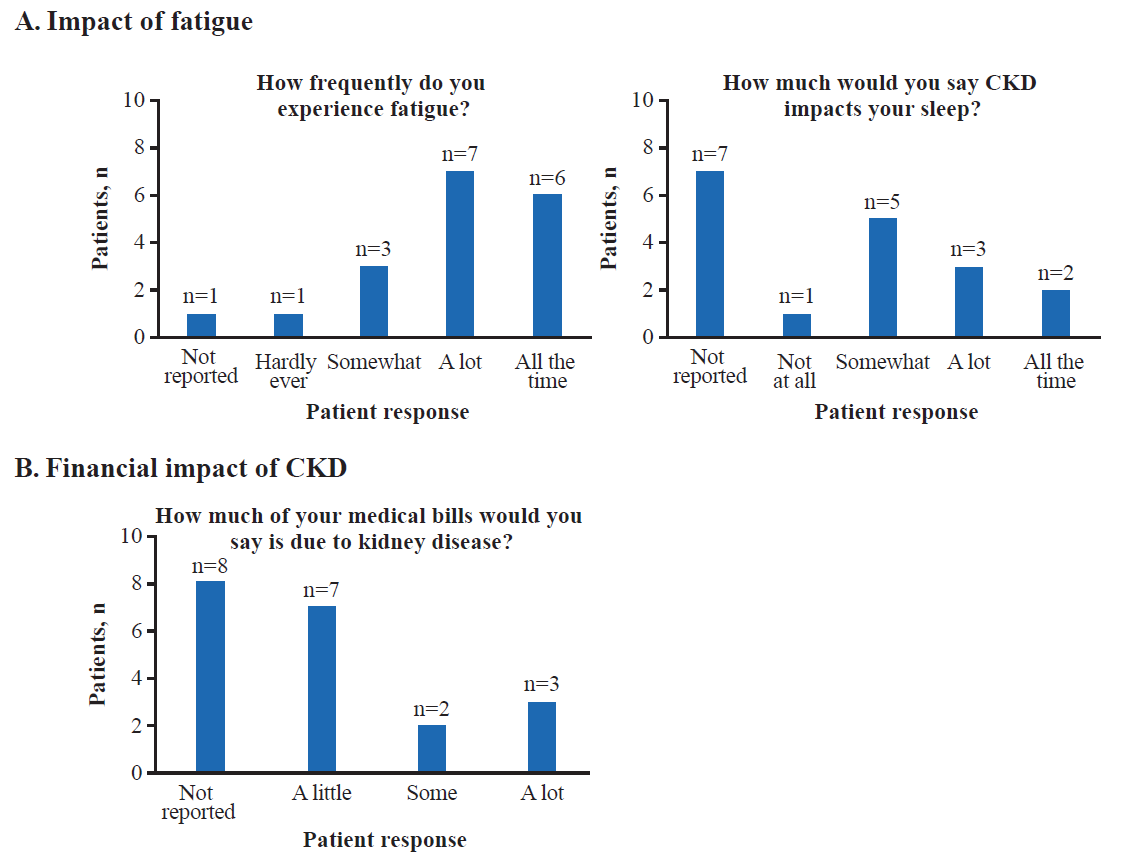
**

CKD, chronic kidney disease.
